# Urine-derived podocytes from steroid resistant nephrotic syndrome patients as a model for renal-progenitor derived extracellular vesicles effect and drug screening

**DOI:** 10.1186/s12967-024-05575-z

**Published:** 2024-08-14

**Authors:** Adele Tanzi, Lola Buono, Cristina Grange, Corinne Iampietro, Alessia Brossa, Fanny Oliveira Arcolino, Maddalena Arigoni, Raffaele Calogero, Laura Perin, Silvia Deaglio, Elena Levtchenko, Licia Peruzzi, Benedetta Bussolati

**Affiliations:** 1 Department of Molecular Biotechnology and Health Sciences, University of Turin, Via Nizza 52, Turin, 10125 Italy; 2 Department of Medical Sciences, University of Turin, Turin, Italy; 3 Department of Pediatric Nephrology, Emma Children’s Hospital, Amsterdam UMC, Amsterdam, The Netherlands; 4 Emma Centrum of Personalized Medicine, Emma Children’s Hospital, Amsterdam UMC, Amsterdam, The Netherlands; 5 Department of Urology, Children’s Hospital Los Angeles, Los Angeles, CA USA; 6 Department of Development and Regeneration, Cluster Woman and Child, Laboratory of Pediatric Nephrology, KU Leuven, Leuven, Belgium; 7 Pediatric Nephrology, ERKNet Center, Regina Margherita Children’s Hospital, AOU Città della, Salute e della Scienza di Torino, Turin, Italy

**Keywords:** Steroid-resistant nephrotic syndrome, Personalized therapy, Exosomes, Permeability, Alport syndrome, Renal progenitor cells, Disease models, SUMOylation, SUMO1, SENP2

## Abstract

**Background:**

Personalized disease models are crucial for evaluating how diseased cells respond to treatments, especially in case of innovative biological therapeutics. Extracellular vesicles (EVs), nanosized vesicles released by cells for intercellular communication, have gained therapeutic interest due to their ability to reprogram target cells. We here utilized urinary podocytes obtained from children affected by steroid-resistant nephrotic syndrome with characterized genetic mutations as a model to test the therapeutic potential of EVs derived from kidney progenitor cells (nKPCs).

**Methods:**

EVs were isolated from nKPCs derived from the urine of a preterm neonate. Three lines of urinary podocytes obtained from nephrotic patients’ urine and a line of Alport syndrome patient podocytes were characterized and used to assess albumin permeability in response to nKPC-EVs or various drugs. RNA sequencing was conducted to identify commonly modulated pathways after nKPC-EV treatment. siRNA transfection was used to demonstrate the involvement of SUMO1 and SENP2 in the modulation of permeability.

**Results:**

Treatment with the nKPC-EVs significantly reduced permeability across all the steroid-resistant patients-derived and Alport syndrome-derived podocytes. At variance, podocytes appeared unresponsive to standard pharmacological treatments, with the exception of one line, in alignment with the patient’s clinical response at 48 months. By RNA sequencing, only two genes were commonly upregulated in nKPC-EV-treated genetically altered podocytes: small ubiquitin-related modifier 1 (SUMO1) and Sentrin-specific protease 2 (SENP2). SUMO1 and SENP2 downregulation increased podocyte permeability confirming the role of the SUMOylation pathway.

**Conclusions:**

nKPCs emerge as a promising non-invasive source of EVs with potential therapeutic effects on podocytes with genetic dysfunction, through modulation of SUMOylation, an important pathway for the stability of podocyte slit diaphragm proteins. Our findings also suggest the feasibility of developing a non-invasive in vitro model for screening regenerative compounds on patient-derived podocytes.

**Supplementary Information:**

The online version contains supplementary material available at 10.1186/s12967-024-05575-z.

## Introduction

Podocytes are highly dynamic and terminally differentiated renal cells that act as the final barrier to proteins during glomerular blood filtration, thus playing a pivotal role in controlling glomerular permeability [1]. Damage to podocytes can result in primary nephrotic syndrome, a prevalent pathology in children characterized by heavy proteinuria, edemas, hypoalbuminemia and hyperlipidemia, with an annual incidence of 2–7 cases per 100,000 children. The standard treatment involves steroid therapy, but around 10% of patients are resistant (steroid-resistant nephrotic syndromes) [2–4] and eventually progress to end-stage kidney disease [5].

The advent of reliable genetic testing has facilitated the identification of causative genetic mutations in one-third of steroid-resistant cases [6]. Glomerular genetic variants affecting structural or secreted podocyte proteins can disrupt multiple podocyte functions. The main mutations involve genes encoding for slit diaphragm proteins (NPHS1 coding for nephrin, and NPHS2 coding for podocin), and for mesangial matrix synthesis (among which COL4A3, COL4A4 and COL4A5, mutated in Alport syndrome and in a percentage of cases of steroid-resistant nephrotic syndrome, as well as LAMB2 coding for laminin subunit beta) [6, 7]. These disruptions can lead to high-grade proteinuria and severe nephrotic syndrome that often progress to a rapid decline in kidney function within a few years [8]. Rate of progression can be forecasted basing on the expected impact of the variant on the protein expression but still, several clinical modifiers (hypertension, response to supportive therapy, other clinical additional risk factors) can influence the individual slope of kidney function decline [9]. For these reasons, in vitro tools to evaluate the response of the single subjects to treatments are highly warranted, with the aim to avoid the multiple attempts to induce proteinuria reduction and the burden of adverse events and toxicity.

Focal detachment of podocytes is a phenomenon observed in cases of massive proteinuria in both experimental [10] and human diseases [11]. Given this, isolating podocytes from urine samples has acquired interest for understanding their pathological characteristics and identifying potential therapeutic responses [12]. In line with this, we recently isolated and characterized podocytes from the urine of patients with Alport syndrome [13].

Extracellular vesicles (EVs), cell-released vesicles involved in cell-to-cell communication, are gaining attention as biological tools with regenerative potential [14]. In renal pathology, EVs derived from stem cells of different sources exert anti-apoptotic, anti-inflammatory, and pro-angiogenic effects, possibly through the transfer of mRNAs, miRNAs, and proteins to renal cells [15–18]. We recent demonstrated the beneficial effect of mesenchymal stromal cell-derived EVs (MSC-EVs) on podocyte injury in an in vitro millifluidic model of the glomerular filtration barrier [19]. Similarly, EVs derived from endothelial were shown to inhibit complement-induced podocyte apoptosis, prevent nephrin shedding, and maintain permselectivity during inflammatory damage [20]. However, the potential effect of stem cell-derived EVs in treating genetically altered podocytes remains unexplored.

In this study, we hypothesized that EVs derived from neonatal kidney progenitor cells (nKPCs) could offer specific benefits to podocytes, given their renal origin. These cells, isolated from preterm neonatal urine were previously reported to express embryonic kidney transcription factors, including SIX2 and CITED1 [21], and were shown to protect renal cells from hypoxic damage in a model of kidney graft perfusion [22]. To explore this hypothesis, we used podocytes with genetic alterations obtained from the urine of children with steroid-resistant nephrotic syndrome and we evaluated the effects and potential mechanisms of nKPC-EVs. These were compared with common drugs known to modulate podocyte permeability. Additionally, we employed urinary conditionally immortalized podocytes from an Alport syndrome patient as a model for genetically diseased podocytes with altered permeability.

## Methods

### Ethical statement

All the enrolled subjects provided informed written consent. The study protocol was approved by the Bioethics Committee of the A.O.U. Città della Salute e della Scienza Hospital (protocol no. 0021671). The study was conducted according to the principles expressed by the Declaration of Helsinki of 1975, as revised in 2013.

### Generation of podocyte cell lines

A total of three patients diagnosed with steroid-resistant nephrotic syndrome were recruited in this study. Steroid resistance was defined according to the global consensus [23] as persistence of proteinuria in nephrotic range without significant reduction from onset after a course of steroid of 4 weeks prednisone 60 mg/m2/day, followed by 3 methylprednisolone pulses of 10 mg/kg each on three consecutive days and two more weeks of observation. If proteinuria is not modified by this treatment the child is classified as “steroid resistant” and is addressed to genetic study. At the time being a panel of 70 genes associated to steroid resistant nephrotic syndrome encoding for proteins involved in podocyte functions is explored [23]. Genetic and clinical features of patients are described in Table 1. ACMG classification refers to the time of genetic diagnosis. During follow up patients 1 and 2 did not respond to all the treatments attempted, progressed to CKD 5 (chronic kidney disease, stage 5) and were successfully transplanted without recurrence. Patient 3, which initially proved to be steroid resistant, had a late satisfactory response to steroids combined with angiotensin-converting enzyme inhibitors and one single dose of Rituximab, and after 48 months of follow up has a normal kidney function and minimal proteinuria (Table 1). As control, we used urine from a healthy pregnant woman, a valuable source for control podocytes due to pregnancy-associated podocyturia [24].

Urine samples (~ 50 ml) were freshly centrifuged at 200 g for 10 min. The pellet was resuspended in DMEM/F-12 (Life Technologies, Thermo Fisher Scientific, Waltham, MA, USA) supplemented with 10% foetal calf serum (FCS; Invitrogen), 50 IU/ml penicillin, 50 g/ml streptomycin, 5 mM glutamine, 5 g/ml insulin, 5 g/ml transferrin, and 5 mg/ml selenium (all from Sigma-Aldrich, St Louis, MO, USA). Subsequently, primary cells were grown at 37 °C up to the third passage and characterized as podocytes. Three lines of steroid resistant nephrotic syndrome podocytes (NS-POD1-3) and a control urine podocyte line (CTL-uPOD) were generated.

In addition, a conditionally immortalized podocyte cell line (AS-POD) from urine of an Alport syndrome patient, previously isolated and characterized in our laboratory, was used [13]. Finally, tissue-derived conditionally immortalized podocytes, which were obtained from a nephrectomy of a healthy subject, were kindly gifted by MA Saleem and used as control for selected experiments (CTL-tPOD) [25]. AS-POD and CTL-tPOD were maintained in culture as previously described [26]. Briefly, cells were grown at a permissive temperature of 33 °C and, 8–10 days before experiments, moved to 37 °C, to ensure growth arrest and differentiation. The culture medium for AS-POD and CTL-tPOD was the same of primary podocyte cell lines.

### Generation of nKPC cell line

Urine sample (500 mL) was collected from a newborn (born at 32 gestational weeks) at day 1 after birth from a catheter bag, as described [21]. The sample was centrifugated at 200 g for 10 min and the cell pellet was resuspended in α-MEM Medium (Gibco, Thermo Fisher Scientific) supplemented with 20% Chang Medium B (Irvine Scientific, Santa Ana, California, USA) and 2% Chang Medium C (Irvine Scientific), 20% FCS (Invitrogen), 50 IU/mL penicillin, 50 g/mL streptomycin, 5 mM glutamine (all from Sigma‐Aldrich). At passage 2, 1 × 10^4^ primary nKPCs were infected in DMEM F12 20% FCS with a retrovirus containing a pBABE-puro-hTERT plasmid (Addgene plasmid #1771) [27]. The day after the infection, the medium was replaced with their growth medium. From passage 3, cells were selected in their growth medium containing 1 µg/mL puromycin (Gibco) for four weeks, until passage 7.

### Non-renal cell lines

Primary human umbilical vein endothelial cells (HUVEC) were purchased from ATCC (ATCC-PCS-100-010, Manassas, VA, USA). Human corneal endothelial cells were previously isolated from discarded cornea patients undergoing corneal transplantation or enucleation and characterized in our laboratory [28]. For HUVEC and corneal endothelial cells, EndoGRO-VEGF (Merck Millipore, Burlington, MA, USA) supplemented with 5% FCS (Invitrogen), was used as culture media. Breast cancer cells were previously isolated, grown in DMEM-F12 supplemented with 10 ng/mL basic fibroblast growth factor, 20 ng/mL epidermal growth factor, 5 µg/mL insulin, and 0.4% bovine serum albumin (all from Sigma-Aldrich), and characterized in our laboratory [29]. Human bone marrow-derived MSCs were purchased from Lonza (Switzerland) and grown in mesenchymal stem cells basal medium (MSCBM, Lonza) [28].

### EV isolation

nKPC-EVs and MSC-EVs were obtained respectively from the supernatant of nKPCs and MSCs cultured overnight in serum free conditions (RPMI 0% FCS), as previously described [19, 28]. Serum-EVs were obtained from a total of 100 mL of serum isolated from a blood pool of five healthy donors, as previously mentioned [30]. Informed consent was obtained by the Blood Bank of “Città della Salute e della Scienza di Torino” from all the donors. Culture supernatant and serum were centrifuged for the removal of cell debris and apoptotic bodies at 3,000 g for 20 min. EVs were then purified by a 2 h-ultracentrifugation at 100,000 g at 4 °C (Beckman Coulter, Brea, CA, USA) and used fresh or stored at − 80 °C after resuspension in RPMI without FSC and supplemented with 1% dimethyl sulfoxide. Analysis of the size distribution and particle quantification were performed using NanoSight NS300 (NanoSight Ltd, Malvern, UK) equipped with a 405 nm laser and the Nanoparticle Tracking Analysis (NTA) 2.3 software (NanoSight Ltd).

### Protein extraction and western blot

For protein analysis, different EV preparations (about 5) were pooled to obtain 10^11^ particles and further ultracentrifuged. Subsequently, the EV pellet was resuspended in lysis buffer, composed as follow: 1:100 Phosphatase Inhibitor Cocktail 2, 1:100 Phosphatase Inhibitor Cocktail 3, 1:100 Phenylmethanesulfonyl fluoride (PMSF), 1:100 Protease Inhibitor Cocktail (all from Sigma-Aldrich), in RIPA buffer (20 nM Tris·HCl, 150 nM NaCl, 1% deoxycholate, 0.1% SDS 1% Triton X-100, pH 7.8, Sigma-Aldrich). After 20 min of incubation at 4 °C, the protein extract was centrifugated for 15 min at 14’000 g and the supernatant was used. Podocyte and nKPC pellets were similarly resuspended in lysis buffer for protein extraction. Protein concentration of podocyte lysate was determined by Bradford solution, according to the manufacturer’s procedures (Bio-Rad Inc, Berkeley, CA, USA). At variance, total protein concentration of nKPCs and nKPC-EVs was determined spectrophotometrically using a micro-BCA™ Protein Assay Kit (Thermo Fisher Scientific), as previously described [31]. Either 30 µg (for podocytes) or 8 µg (for nKPCs and nKPC-EVs) of proteins were electrophoresed through 4–12% Mini-Protean TGX Stain-Free Gels (Bio-Rad). Using the iBLOT2 system (Life Technologies, Carlsbad, CA, USA), gels were blotted onto PVDF membrane filters according to the manufacturer’s procedures. Each membrane was incubated with blocking solution, consisting in 5% bovine serum albumin (BSA; Sigma-Aldrich) in PBS, for 1 h before overnight incubation with primary antibodies at the indicated dilutions. After rinsing in wash buffer (0.1% Tween in PBS), horseradish peroxidase-conjugated secondary antibodies were used for 1 h at 1:3000–1:5000 dilutions. After final washings, membranes were incubated with ECL chemiluminescence reagent (Bio-Rad, Milan, Italy). Images were acquired using a ChemiDoc ™ XRS + System (Bio-Rad). For podocytes analysis, the following antibodies were used: rabbit monoclonal anti-Podocin (Cat. No. sc-21009; Santa-Cruz, Dallas, TX, USA), and mouse monoclonal anti-CD2AP (Cat. No. sc-25272, Santa-Cruz). Goat monoclonal anti-Vinculin (Cat. No. sc-7648, Santa-Cruz) and mouse monoclonal anti-PCNA (Cat. No. sc-56, Santa-Cruz) were used as housekeepings. In the case of nKPC and nKPC-EV protein analyses, mouse monoclonal anti-CD63 (Cat. No. sc-5275, Santa-Cruz), rabbit monoclonal anti-Calreticulin (Cat. No. 2891, Cell signalling, Milan, Italy) and mouse monoclonal anti-TSG101 (Cat. No. sc-7964, Santa-Cruz) antibodies were used. The protein bands were detected using either rabbit, mouse, or goat peroxidase-labeled secondary antibodies.

### Immunofluorescence

Immunofluorescence on podocytes was performed as follows: cells were plated at a density of 4 × 10^4^ cells/cm^2^ and the next day fixed in 4% paraformaldehyde for 20 min at room temperature and permeabilized with PBS 0.1% Triton X-100 (Sigma-Aldrich) for 10 min at 4°C. PBS 1.5% BSA (Sigma-Aldrich) was used to block non-specific sites for 20 min at room temperature. Subsequently, Texas Red-X Phalloidin (Cat. No. T7471, Thermo Fisher Scientific) was incubated for 1 h. Fixed cells were washed with PBS 0.1% BSA before nuclear staining with 4.6-diamidine-2-phenylindole (DAPI, Sigma-Aldrich) for 8 min. After the final wash, coverslips were mounted with Fluoromount (Sigma-Aldrich). Images were acquired by the videoconfocal system ViCo microscope Nikon Eclipse 80i (Nikon, Japan).

### Transmission electron microscopy

The transmission electron microscopy (TEM) was performed on EVs fixed in glutaraldehyde placed on 200-mesh nickel formvar carbon-coated grids (Electron Microscopy Science) for 20 min to promote adhesion. The grids were then incubated with 2.5% glutaraldehyde plus 2% sucrose. EVs were negatively stained with NanoVan (Nanoprobes, Yaphank, NY, USA) and observed using a Jeol JEM 1400 Flash electron microscope (Jeol, Tokyo, Japan) [32].

### Super-resolution microscopy

Super-resolution microscopy was performed with Nanoimager S Mark II microscope from ONI (Oxford Nano-imaging, Oxford, UK) equipped with a 100x, 1.4NA oil immersion objective, an XYZ closed-loop piezo 736 stage, and triple emission channels split at 640, 555 and 488 nm on nKPC-EV. EV profiler Kit (EV-MAN-1.0, ONI) was used for the experiments following manufacturer’s protocol. The Kit contains fluorescent antibodies, anti CD9-488, CD63-568 and CD81-647, washing buffer and the imaging buffer. Images were acquired sequentially in dSTORM mode in total reflection fluorescence (TIRF). Single-molecule data was filtered using NimOS software (v.1.18.3, ONI). Data analysis was conducted using Collaborative Discovery (CODI) online analysis platform www.alto.codi.bio from ONI and the drift correction pipeline version 0.2.3 was used [33].

### RNA isolation, real time PCR and RNA sequencing analysis

Total RNA of patient-derived podocytes untreated or treated for 24 h with nKPC-EVs or MSC-EVs (5 × 10^4^ EVs/cell), as well as RNA of CTL-tPOD untrasfected or transfected with siRNAs, was isolated using Trizol Reagent (Ambion, Austin, TX, USA) according to the manufacturer’s protocol. At variance, RNA of nKPCs and of nKPC-EVs was extracted using miRNeasy mini kit (Qiagen GmbH, Hilden, Germany) according to the manufacturer’s protocol. RNA was then quantified spectrophotometrically (Nanodrop ND-1’000, Wilmington, NC, USA). For the gene expression analysis, quantitative real-time PCR (RT-PCR) was performed. Briefly, one-strand cDNA was produced from 200 ng of total RNA using a High-Capacity cDNA Reverse Transcription Kit (Applied Biosystems, Waltham, MA, USA). RT-PCR experiments were performed in a 20 µL-reaction mixture containing 5 ng of cDNA template, the sequence-specific oligonucleotide primers (purchased from MWG-Biotech, Eurofins Scientific, Brussels, Belgium), and the Power SYBR Green PCR Master Mix (Applied Biosystems). GAPDH mRNA was used to normalize the RNA inputs. The fold change expression with respect to the control was calculated for all the samples. Primer list can be found in supplementary data (Supplementary table 1). For the RNA sequencing analysis, libraries for RNA-seq were generated using a TruSeq RNA stranded sample preparation kit v2 (Illumina Inc, San Diego, CA, USA) following the manufacturer’s instructions, using 1 µg of total RNA as input material. Libraries were pooled and sequenced with a NextSeq 500 sequencer (Illumina Inc) generating 75-bp paired-end sequences. Further analyses were performed using transcript per million (TPM) tables and genes with an average |log_2_ Fold Change| ≥1 were considered for further analysis using Expression Suite and Funrich V3 Software (Bundora, Australia).

### Flow cytometry

After puromycin selection, nKPCs were detached using a nonenzymatic cell dissociation solution, resuspended in PBS 0.1% BSA (Sigma-Aldrich) and incubated with antibodies. Cells were incubated with either phycoerythrin (PE)-, fluorescein isothiocyanate (FITC)-, or allophycocyanin (APC)-conjugated antibodies against CD90 (Cat. No. 130-114-859, Miltenyi Biotec, Bergisch Gladbach, Germany) CD73 (Cat. No. 550257, BD Bioscience, Franklin Lakes, NJ, USA), CD146 (Cat. No. 550315, BD Bioscience) and CD29 (Cat. No. 130-101-256, Miltenyi Biotec) CD133-1 (Cat. No. 130-090-826, Miltenyi Biotec) and appropriate isotype control. Stained cells were then analyzed using FACSCalibur machine using CellQuest software (Becton Dickinson Bioscience Pharmingen).

### MACSPlex analysis

nKPC-EVs were subjected to bead-based multiplex EV analysis by flow cytometry (MACSPlex Exosome Kit, human, Miltenyi Biotec) as previously described [33]. Briefly, 5 × 10^9^ EVs were diluted with a MACSPlex buffer (MPB) to a final volume of 120 µL and 15 µL of MACSPlex Exosome Capture Beads (containing 39 different antibody-coated bead subsets) were added to each sample. The samples were then incubated on an orbital shaker overnight (14–16 h) at 450 rpm at 4 °C protected from light, followed by several washings with MPB using centrifugations (3’000 g, 5 min). For EV counterstaining, 5 µL of each APC-conjugated anti-CD9, anti-CD63, and anti-CD81 detection antibodies were added to each sample and incubated on an orbital shaker at 450 rpm for 1 h at room temperature. After additional washings, samples were subjected to flow cytometric analysis using FACS Celesta (BD Biosciences, New Jersey, USA).

### Permeability assay

Permeability assays were performed in a 24 well plate using cell culture inserts with a pore size of 0.4 μm (Corning, New York, USA). Four x 10^4^ podocytes were plated on the insert and left adhere overnight. Subsequently, podocytes were treated for 24 h with nKPC-EVs (2–5 × 10^4^ EVs/cell), methylprednisolone (Urbason, Sanofi, 40 µg/mL), cyclosporin (Sandimmun, Novartis, 25 µg/mL), tacrolimus (Prograf, Panacea Biotec, 1 ng/mL) or rituximab (Mabthera, Roche, 12 µg/mL). Subsequently, FBS-free medium (500 µL) containing or not FITC-BSA (1 mg/mL, Sigma-Aldrich) was placed in the lower compartment and upper podocyte compartments, respectively. After 6 h, BSA filtration from the lower to the upper compartment was measured, to evaluate the podocyte filtration ability in basal to apical direction. Therefore, 100 µl of medium was taken from the upper compartment and the passage of FITC-BSA was determined by fluorimetry in triplicate, using Promega™ GloMax^®^ Plate Reader (Promega Italia S.r.l., Milano, Italy). Data are expressed as the mean amount of filtered BSA-FITC of four different experiments using at least three inserts for each condition in each experiment.

### Podocyte transfection

Transfection of CTL-tPOD was performed using Lipofectamine RNAiMAX Reagent (Invitrogen). Briefly, 4 × 10^4^ CTL-tPOD/well were plated in cell culture inserts, as described for the permeability assay, left adhere overnight at 33 °C and therefore moved at 37 °C, to promote differentiation. After 8 days at 37 °C, cells were transfected with 1.2 µl of Lipofectamine and 10 picomol of the specific MISSION esiRNAs (SUMO1 and SENP2; Merck), according to the manufacturer’s instructions. The day after transfection, fresh growth medium was replaced and 48 h later the cells were used for the permeability assay. In parallel, transfected CTL-tPOD were lysed, RNA was extracted, and RT-PCR was performed to verify downregulation of SUMO1 and SENP2 mRNAs. Cells transfected with MISSION negative control (Merck) were used as control.

### Statistical analysis

Data are shown as mean ± SD. Statistical analysis was carried out on Graph Pad Prism (GraphPad Software, Inc., San Diego, CA, USA) by using one-way analysis of variance (ANOVA) followed by Dunnet’s multiple comparisons test, or by unpaired t-test, where appropriate. A p value < 0.05 was considered significant.

## Results

### Podocyte isolation and characterization

Podocytes were obtained from freshly collected urine derived from three different patients presenting steroid-resistant nephrotic syndrome (NS-POD). Genetically characterized mutations are described in Table 1. Podocytes isolated from healthy pregnant woman’s urine were used as control (CTL-uPOD). Additionally, conditionally immortalized podocytes, isolated from the urine of a patient with Alport syndrome and previously characterized in our laboratory [13], were included in the study (AS-POD) (Table 1).

**Table 1:** Genetic and clinical features of patients

Patient	Sex	Gene	Variant	Zygosity	ACMG classification	Coded protein	Clinical follow-up after 48 months
NS-POD1	M	*NPHS2*	c.479 A > G	Compound Heterozygous -	C4	Podocin	Progression to CKD stage 5 and transplant
			c.855_856delAA		C5	Podocin	
NS-POD2	M	*PLCE1*	c.1405T > A	Compound Heterozygous -	C1	Phospholipase C epsilon 1	Progression to CKD stage 5 and transplant
			C.3281G > C		C3		
NS-POD3	F	LAMB2	c.4874_4876delTCC	Heterozygous	C3	Laminin beta-2 subunit	Clinical remission with proteinuria/creatininuria: < 0.3 mg/mg
AS-POD	F	COL4A3	c.2777del	Heterozygous	C4	Collagen alpha 3	Progression to CKD stage 5 and transplant

*Abbreviations* NS-POD: nephrotic syndrome podocytes; F: female; M: male; NPHS2: Stomatin Family Member, Podocin gene; PLCE1: Phospholipase C epsilon 1 gene; LAMB2: Laminin beta-2 subunit gene; COL4A3: Collagen alpha 3 gene; CKD: chronic kidney disease. ACMG: American College of Medical Genetics and Genomics, ACMG classification refers to the time of genetic diagnosis

**Fig. 1:**
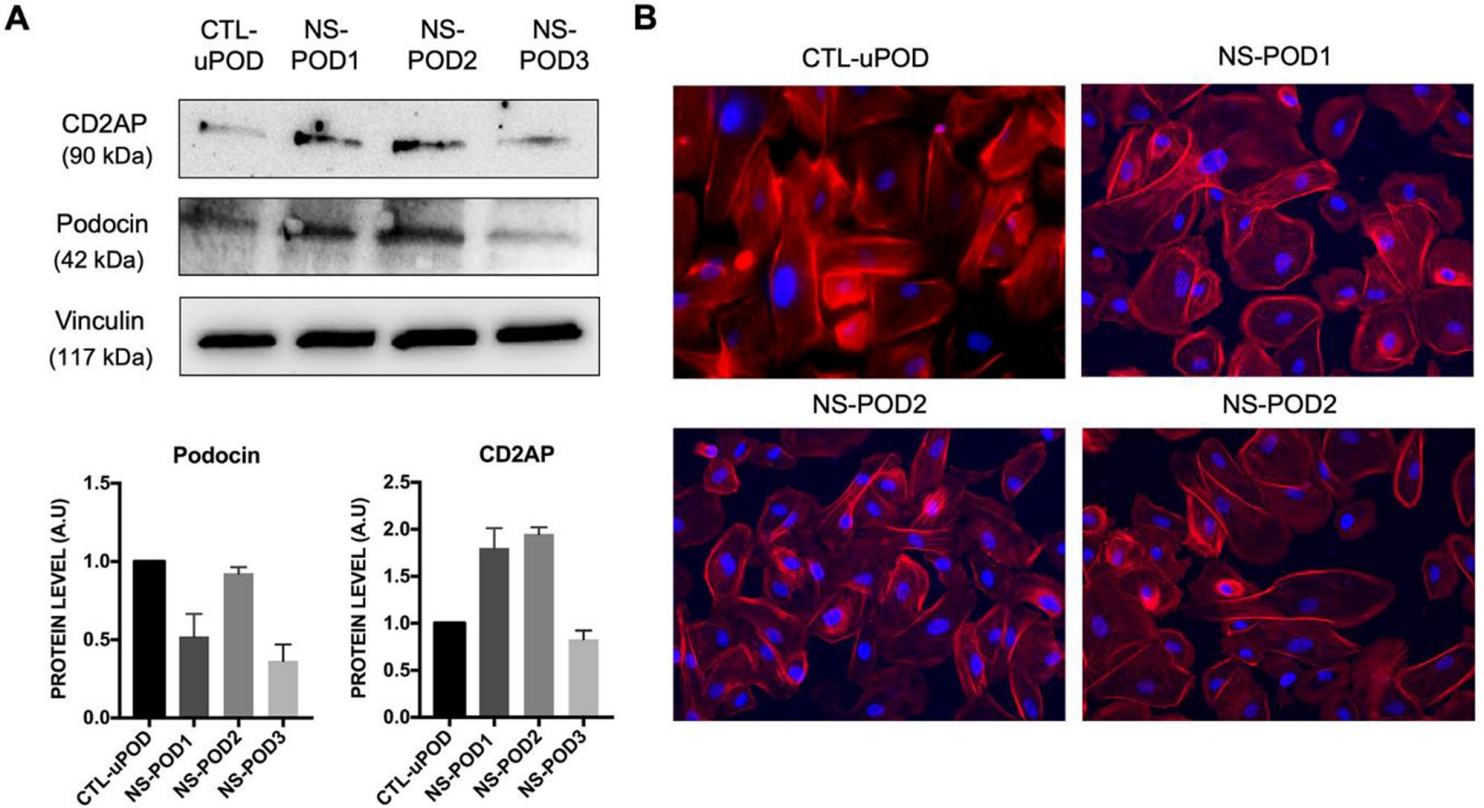
Isolation and characterization of podocytes from urine. (**A**) Western Blot analysis (representative images and quantification) of podocytes derived from urine of three different patients (NS-POD1, NS-POD2, NS-POD3) and of a healthy pregnant woman (CTL-uPOD) positive for CD2AP and podocin. Vinculin was used as housekeeping. Data are expressed as mean of two experiments ± SD. (**B**) Representative micrographs of podocytes deriving from urine of CTL-uPOD, NS-POD1, NS-POD2 and NS-POD3 stained with phalloidin (red) and blue nuclear stain DAPI. Original magnification: X20

Urine-derived cells were characterized by the presence of podocyte markers such as podocin and CD2AP (Fig. 1A). By morphology, cells showed an organized cytoskeletal structure similar to already well-characterized podocytes (Fig. 1B) [13]. Non-renal cell appeared negative for podocytes’ markers (Supplementary Fig. 1).

The podocyte phenotype was also assessed by analyzing the expression of a specific signature composed of 68 podocyte-typical genes (Supplementary Fig. 2), as previously outlined by Lu et al., [13, 34]. All three podocyte lines expressed the typical podocyte genes, thereby confirming the podocyte phenotype (Supplementary Fig. 2) [13].

### Kidney progenitor-derived EV isolation and characterization

**Fig. 2:**
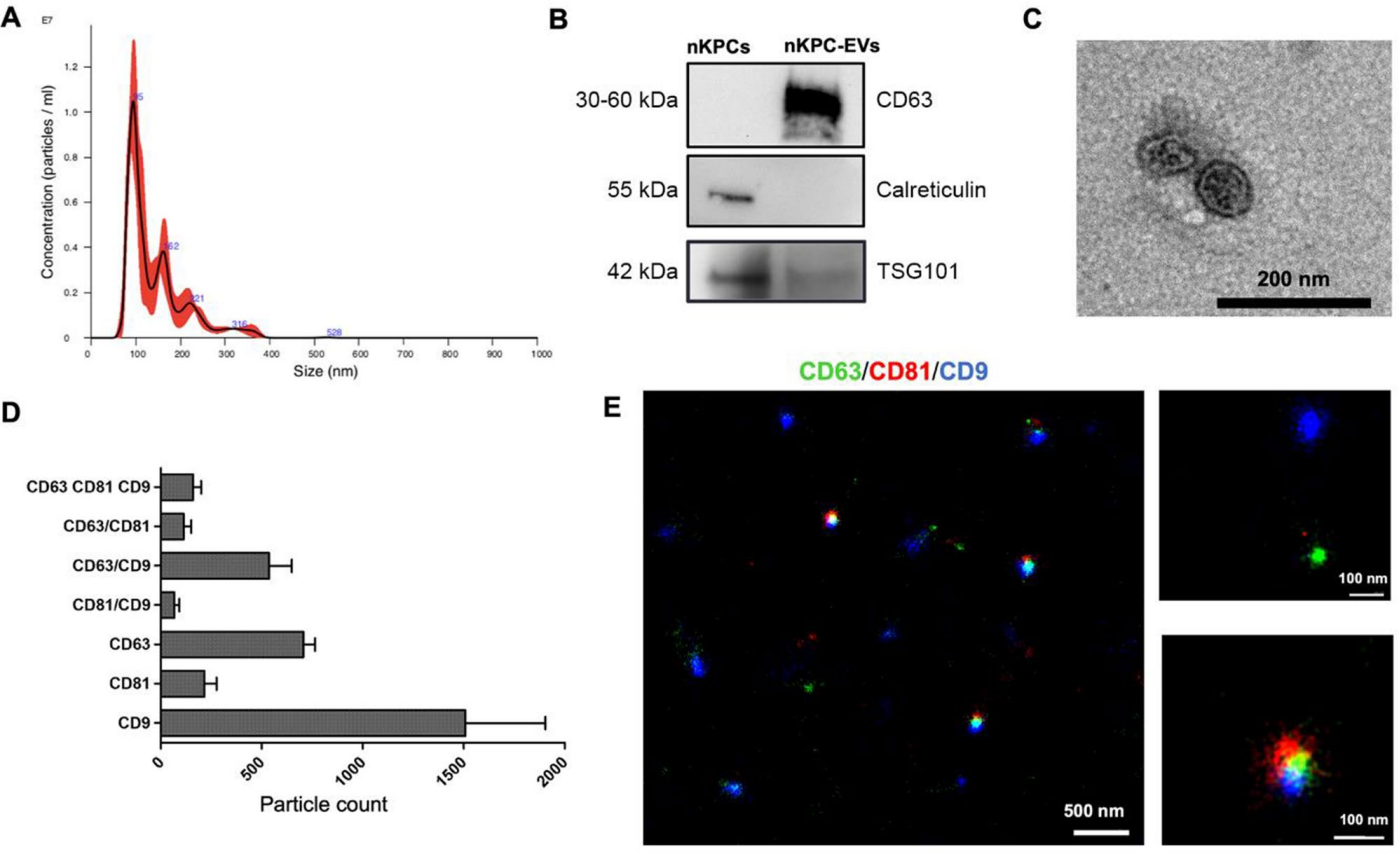
Characterization of nKPC-EVs. (**A**) Representative NTA analysis showing the EV size distribution; EV mean size is about 142.7 nm. (**B**) Representative Western Blot images showing the presence in nKPC-EVs of CD63 and TSG101, and the absence of calreticulin. (**C**) Representative micrograph of TEM of nKPC-EVs (scale bar: 200 nm). (**D**) Clustering analysis of super-resolution microscopy images shows the single, double, and triple positive EV fractions expressing the tetraspanins (CD9, CD63, CD81). The analyses were performed in 3 nKPC-EV preparations using a CODI software; the graph shows the mean ± SD of a cumulative analysis of 3 fields for each preparation. (**E**) Representative super-resolution microscopy images of nKPC-EVs showing expression of CD63 (green), CD81 (red) and CD9 (blue). The scale bars are below each EV image

EVs were isolated by differential centrifugations from immortalized nKPCs. These cells were originally obtained from the urine of a preterm infant and characterized as previously described [21]. The size distribution of nKPC-derived EVs was analyzed using nanosight tracking analysis (NTA), revealing a mean size distribution of approximately 142.7 nm (Fig. 2A). Western Blot analysis confirmed the presence of the EV-specific markers CD63 and TSG101, while the absence of the cytoplasmic marker calreticulin indicated the absence of cellular contamination (Fig. 2B). TEM image revealed the typical cup-shaped morphology of EVs (Fig. 2C). Additionally, super-resolution microscopy demonstrated that nKPC-EVs expressed CD9, CD63, and CD81, typical EV markers, in various combinations, predominantly being CD63 and CD9 single positive (Fig. 2D-E).

**Fig. 3:**
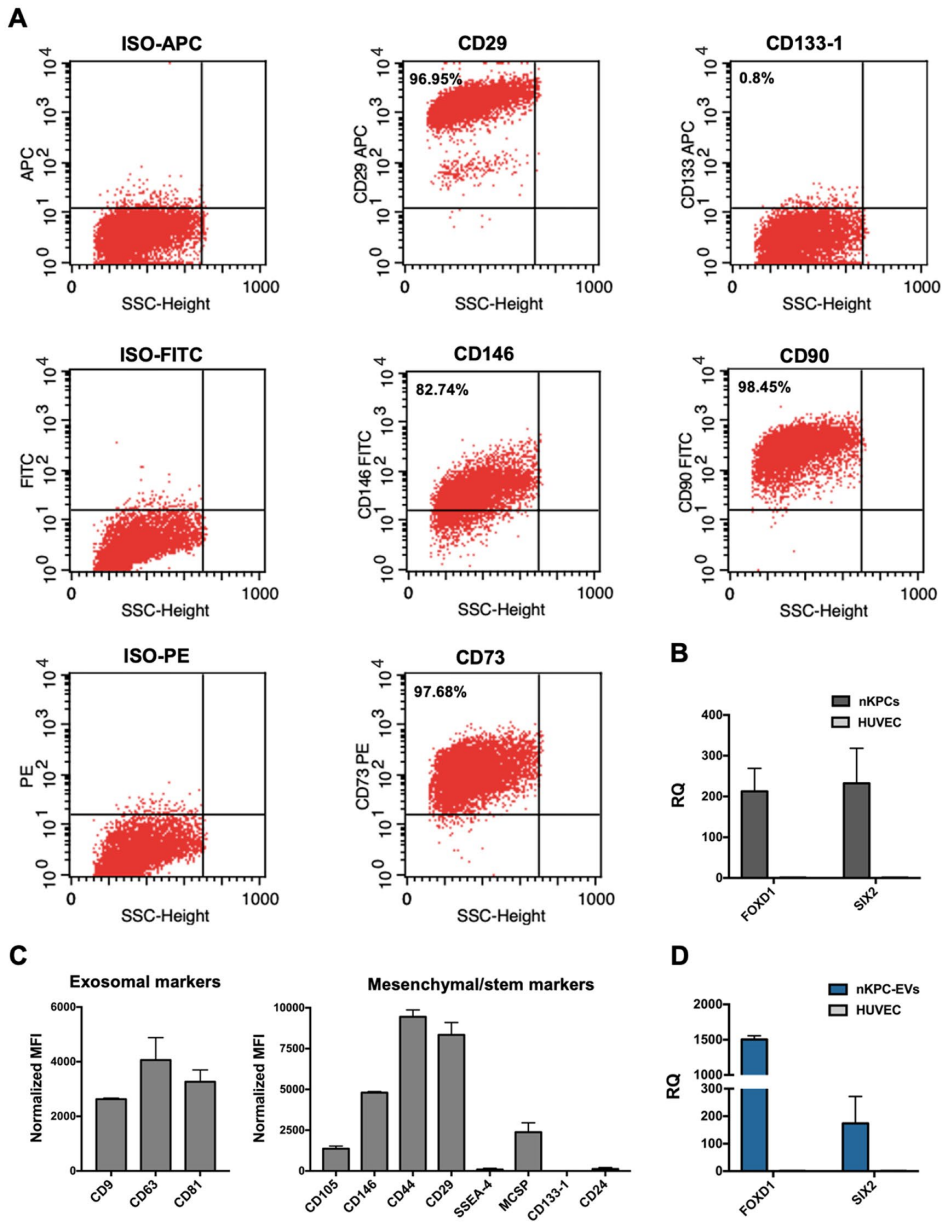
nKPC and nKPC-derived EV characterization of intracellular and surface markers expression. (**A**) Representative flow cytometry dot plots of nKPCs showing positive expression of CD73, CD90, CD29 and CD146; nKPCs resulted negative for CD133 marker. (**B**) RT-PCR analyses showing the expression of FOXD1 and SIX2 in nKPCs. GAPDH was used as endogenous normalizer. Data were further normalized to HUVEC cells, used as a negative control for each experiment. The graphs show the RQ average (2^−∆∆Ct^) of three independent experiments ± SD. (**C**) Quantification of the median APC fluorescence for each bead population after background correction of exosomal and mesenchymal/stem cell markers in nKPC-EVs. The fluorescence intensity of each marker was normalized to the mean fluorescence intensity of all detectable markers to 1000. Data are expressed as the average of two technical replicates ± SD. (**D**) RT-PCR analysis showing the expression of FOXD1 and SIX2 in nKPC-EVs. GAPDH was used as endogenous normalizer. Data were further normalized to HUVEC cells, used as a negative control for each experiment. The graphs show the RQ average (2^−∆∆Ct^) of two independent experiments ± SD

The expression of mesenchymal and renal progenitor markers in nKPCs and deriving EVs was also evaluated. Notably, nKPCs exhibited a mesenchymal phenotype, expressing CD90, CD73, CD146, and CD29 (Fig. 3A), along with the cytoplasmatic nephron progenitor marker SIX2 and stromal progenitor marker FOXD1 (Fig. 3B), as previously reported [21]. Conversely, nKPCs were negative for markers associated with adult renal progenitor cells, such as CD133 (Fig. 3A) [35]. Similarly, using a bead-based immunocapture assay, nKPC-EVs were found to express mesenchymal stromal markers CD146, CD29, and CD44, in addition to the typical EV markers (CD9, CD63, and CD81). At variance, no expression of CD133 or stage-specific embryonic antigen-4 (SSEA-4) was detected (Fig. 3C). Furthermore, nKPC-EVs expressed SIX2 and FOXD1 mRNAs, as demonstrated by RT-PCR, as the originating cells (Fig. 3D).

### Permeability analysis of podocyte cultures

**Fig. 4:**
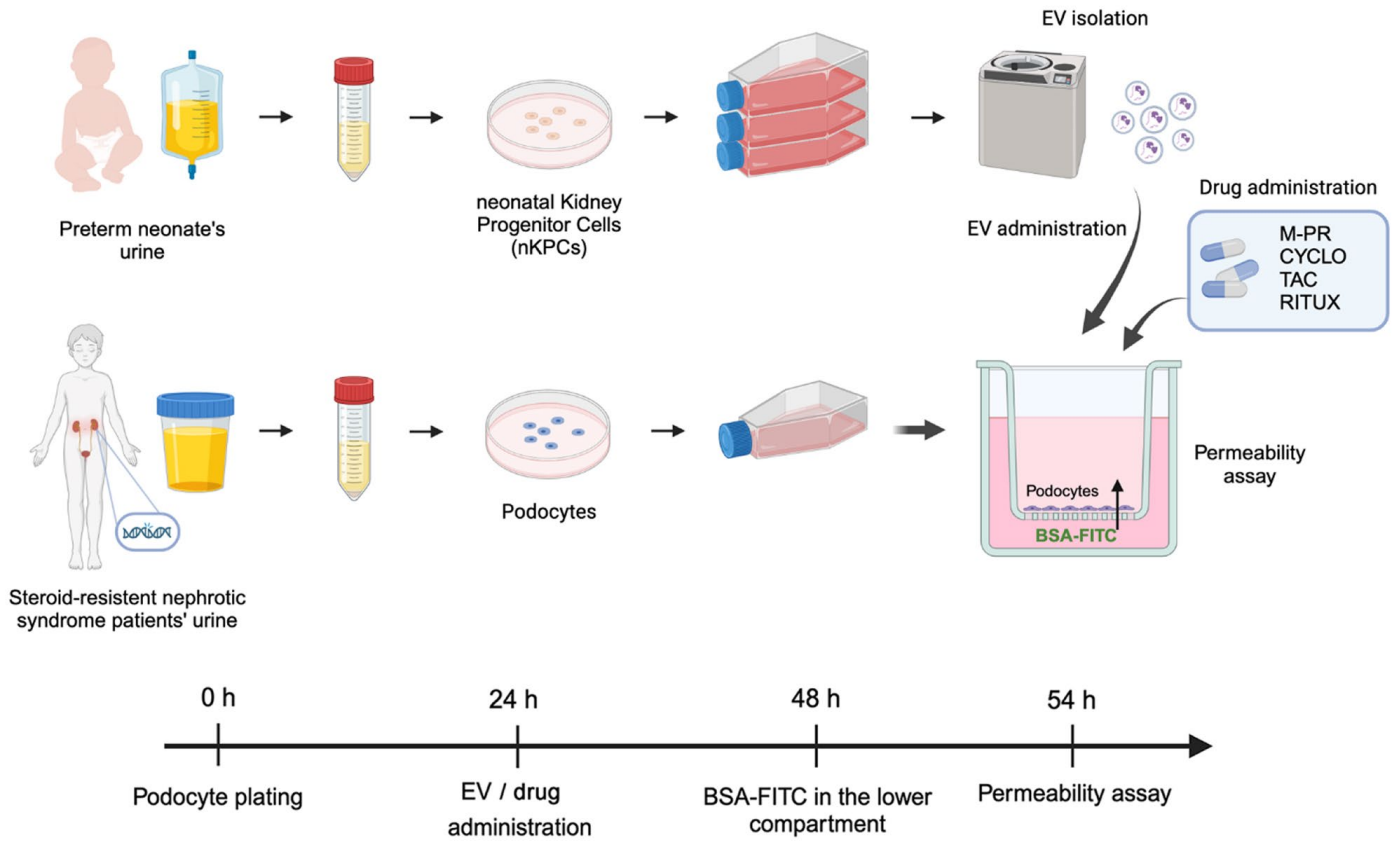
Schematic experimental design and graphic representation of culture set-up. Graphical representation of nKPC and patient-derived podocyte isolation and EV production. Podocytes were plated in the upper compartment of transwell inserts and treated with EVs or drugs. For the permeability assay, the BSA-FITC filtration was measured. *Abbreviations* NS-POD: nephrotic syndrome podocytes; AS-POD: Alport syndrome podocytes; CTL-uPOD: control urinary podocytes; M-PR: methylprednisolone; CYCLO: cyclosporin; TAC: tacrolimus; RITUX: rituximab. The figure was created with Biorender.com

To evaluate whether nKPC-EVs could modulate permselectivity of the patient-derived podocytes, we used an in vitro permeability assay. Podocytes were seeded on the upper side of inserts and permeability was assessed by measuring the transit of FITC-BSA from the lower compartment to the upper podocyte compartment as described in the graphical representation (Fig. 4).

**Fig. 5:**
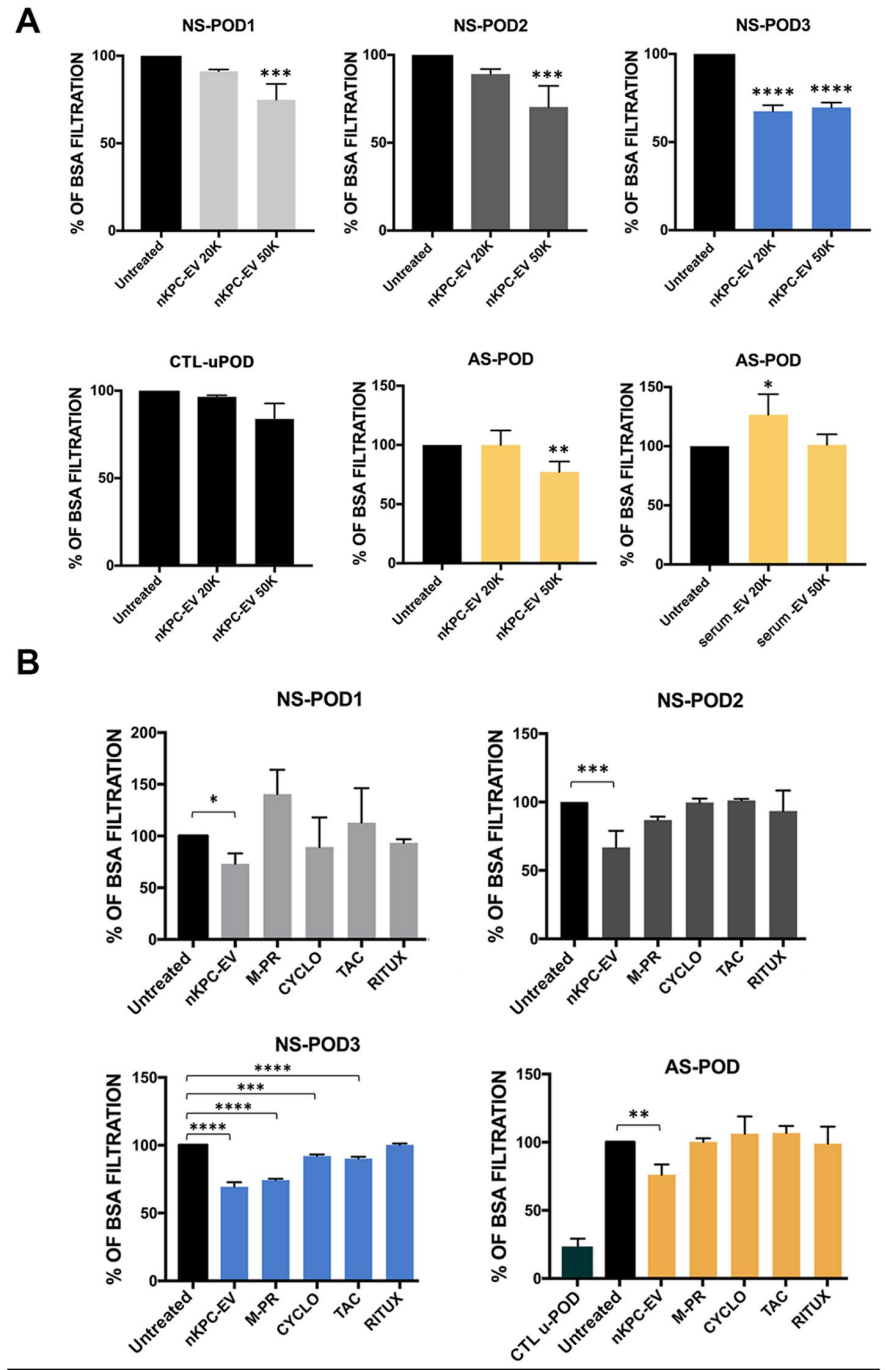
Permeability assay in podocyte cultures treated with nKPC-EVs and different drugs. (**A**) Podocytes were treated with different doses of nKPC-EVs (20 K: 2 × 10^4^ EVs/cell, 50 K: 5 × 10^4^ EVs/cell). Serum-EVs were used as control. Untreated cell condition was used as control for each experiment, set as BSA filtration rate of 100%. Data are expressed as the mean amount of filtered BSA-FITC of four different experiments using at least three inserts for each condition in each experiment ± SD. One-way ANOVA with Dunnett’s multiple comparisons test was performed after the normalization of each experiment to untreated podocytes; * *p* < 0.05, ** *p* < 0.01, *** *p* < 0.001, **** *p* < 0.0001, vs. the respective untreated condition. (**B**) Podocytes were treated with nKPC-EVs (5 × 10^4^ EVs/cell) or the different drugs methylprednisolone (M-PR, 40 µg/mL), cyclosporin (CYCLO, 25 µg/mL), tacrolimus (TAC 1 ng/mL) and rituximab (RITUX 12 µg/mL). Untreated cell condition was used as control for each experiment, set as BSA filtration rate of 100%. Data are expressed as the mean amount of filtered BSA-FITC of four different experiments using at least three inserts for each condition in each experiment ± SD. One-way ANOVA with Dunnett’s multiple comparisons test was performed after the normalization of each experiment to untreated podocytes; * *p* < 0.05, ** *p* < 0.01, *** *p* < 0.001, **** *p* < 0.0001, vs. the respective untreated condition

To evaluate the efficacy of nKPC-EV administration in the modulation of podocyte permeability, experiments were performed using two different EV doses (2 × 10^4^ and 5 × 10^4^ EVs/cell). A significant reduction of permeability was observed using the 5 × 10^4^ EVs/cell dose on all NS-POD lines, as well as on AS-POD (Fig. 5A). No significant changes were observed on control urine podocytes (CTL-uPOD) (Fig. 5A). The reduction of permeability was lacking when serum-EVs were used on AS-POD. Moreover, nKPC-EV effect was compared to that of commonly used drugs for treating renal diseases: methylprednisolone (M-PR), cyclosporin (CYCLO), tacrolimus (TAC), and rituximab (RITUX). Indeed, all these drugs have been reported to exert a direct anti-proteinuric action on podocytes, beside immunomodulation [36–38]. Drug treatment did not affect podocyte viability (Supplementary Fig. 3). No modulation of permeability by pharmacological treatments was observed in the genetically altered podocytes, except for NS-POD3 (Fig. 5B). Interestingly, the patient generating NS-POD3 cells clinically responded to treatment with steroids, cyclosporin and rituximab, showing at 48 months a clinical remission (see methods and Table 1). Permeability of normal podocytes (CTL-uPOD) was significantly lower in respect to diseased podocytes (Fig. 5B), as described [13].

### Transcriptomic analysis of urine-derived podocytes treated with EVs

**Fig. 6:**
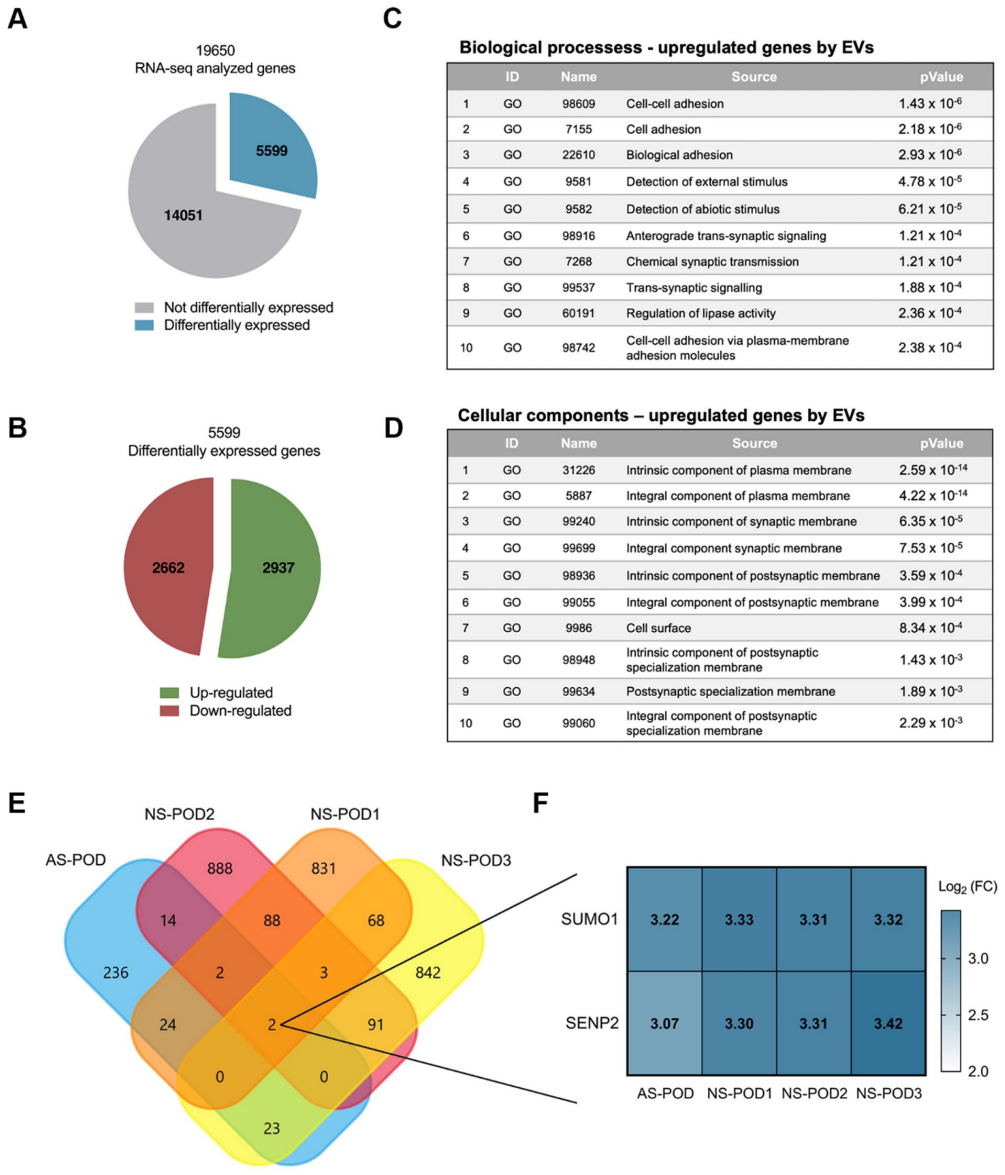
Cross-analysis for the identification of regulated genes common to the four patient-derived podocytes. (**A**) Pie chart representing the sum of all the genes differentially expressed in the four different podocyte lines after the treatment with nKPC-EVs (in blue), compared with the non-differentially expressed genes (grey). (**B**) Pie chart representation of up-regulated (green) and down-regulated (red) differentially expressed transcripts in the four different podocyte lines untreated and treated with EVs. **C** and **D**. Gene Ontology analysis of differentially expressed genes in the four different podocyte lines untreated and treated with EVs. In each table, the identification (ID) number, the name, and the P value associated with the GO are given. **E**. Representative Venn diagram showing the numbers of the genes that resulted up-regulated after the treatment of the podocytes with nKPC-EVs by total RNA sequencing analysis. Data were analyzed using Expression Suite and Funrich V3 Software. **F**. Heatmap showing the levels of expression regulation of the two genes which were upregulated in all the four podocyte lines

To explore the mechanism involved in the observed effect on permeability, we analyzed the change in the RNA profile of the NS-POD and AS-POD after treatment with 5 × 10^4^ nKPC-EVs/cell. The transcripts per kilobase million (TPM) normalization method was employed to compare changes in the gene expression profile between the samples. Our analysis revealed a total of 2876 upregulated genes (log_2_ fold change > 1) and 2574 downregulated genes (log_2_ fold change <-1) in response to EV treatment (Fig. 6A-B). Gene Ontology analysis highlighted cell-to-cell adhesion as the primary upregulated biological activity in treated podocytes (Fig. 6C-D). Only two genes, SUMO1 and SENP2, resulted to be commonly upregulated in all four samples post nKPC-EV treatment (Fig. 6F), showing a log_2_ fold change ranging between 3.07 and 3.42 (Fig. 6E-F). At variance, no gene was consistently downregulated in all patient-derived podocyte cell lines (not shown). Interaction network analysis demonstrated a direct interaction between these two genes in the SUMOylation pathway [39, 40].

**Fig. 7:**
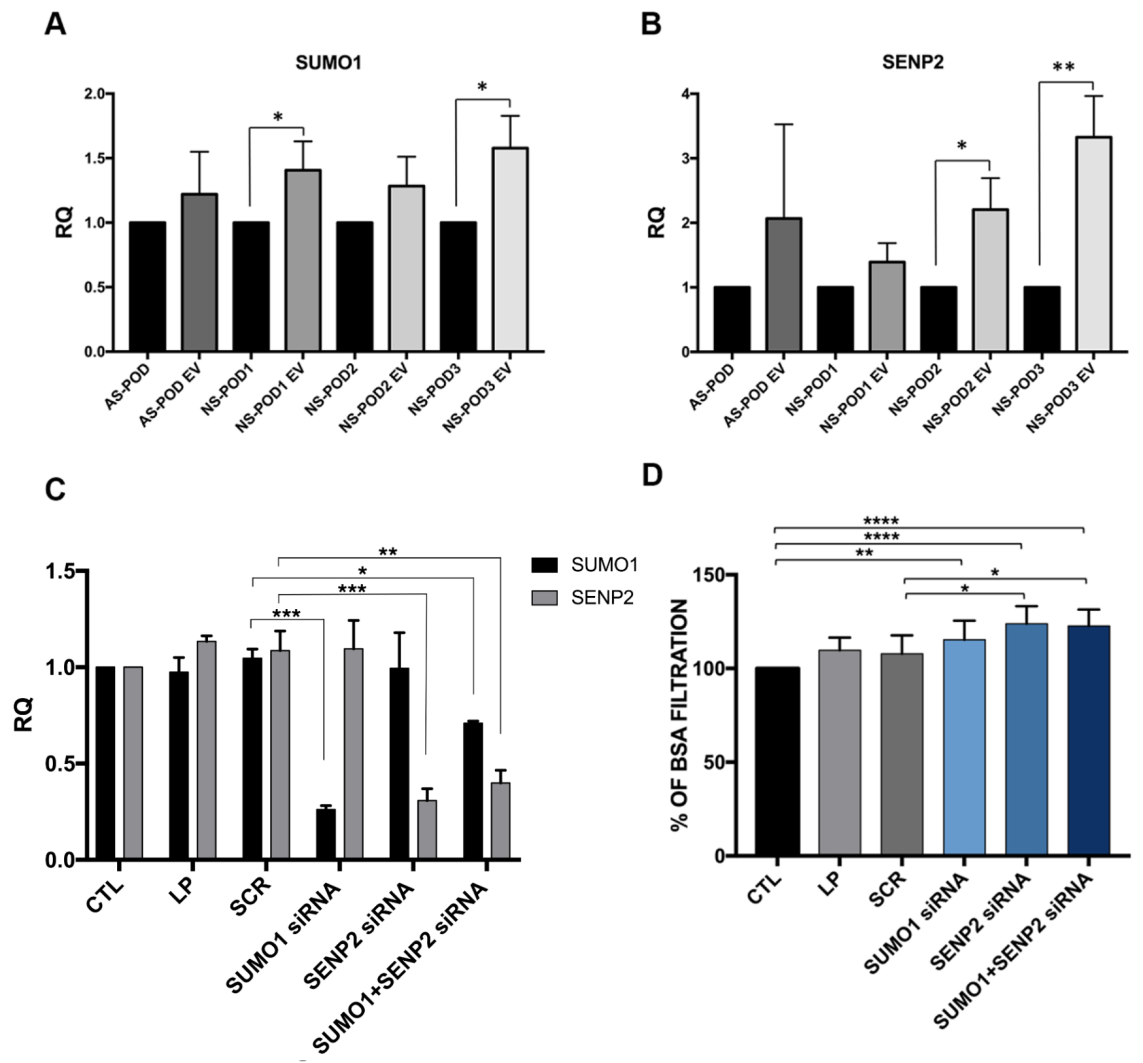
Role of SUMO1 and SENP2 in podocyte permeability. **A**-**B**. Validation of differentially expressed genes in urine-derived podocytes treated with EVs. mRNA expression of SUMO1 (**A**) and SENP2 (**B**) genes in urine-derived podocytes treated or not with nKPC-EVs. Data are shown as relative quantification, normalized to GAPDH and to each untreated control respectively set as 1. The graphs show the RQ average (2^−∆∆Ct^) of at least three independent experiments ± SD. Unpaired t-test was performed after the normalization of each experiment to its untreated condition (AS-POD, NS-POD1, NS-POD 2, NS-POD3, respectively); * *p* < 0.05, ** *p* < 0.001, *** *p* < 0.0001 vs. AS-POD, NS-POD1, NS-POD 2, NS-POD3, respectively. **C**. RT-PCR analysis of SUMO1 and SENP2 genes in CTL-tPOD after siRNA transfection. Data are shown as relative quantification, normalized to GAPDH and to the untransfected control (CTL), set as 1. The graphs show the RQ average (2^−∆∆Ct^) of two independent experiments ± SD. One-way ANOVA with Dunnett’s multiple comparisons test was performed; * *p* < 0.05, ** *p* < 0.01, *** *p* < 0.001, vs. SCR. **D**. Permeability assay using CTL-tPOD transfected with siRNA targeting SUMO1 and/or SENP2 genes. Data are expressed as the mean amount of filtered BSA-FITC of at least six different experiments for each condition. One-way ANOVA with Dunnett’s multiple comparisons test was performed after the normalization of each experiment to untransfected podocytes (CTL); * *p* < 0.05, ** *p* < 0.01, *** *p* < 0.001, **** *p* < 0.0001, vs. SCR. *Abbreviations* CTL: untransfected CTL-tPOD; LP: CTL-tPOD treated only with Lipofectamine reagent; SCR: CTL-tPOD transfected with a scramble sequence; SUMO1 siRNA: CTL-tPOD transfected with siRNA targeting SUMO1; SENP2 siRNA: CTL-tPOD transfected with siRNA targeting SENP2; SUMO1 + SENP2 siRNA: CTL-tPOD transfected with the combination of siRNA targeting SUMO1 and siRNA targeting SENP2

The nKPC-EV-induced up-regulation of both SUMO1 and SENP2 in urine-derived podocytes was confirmed by RT-PCR, where the up-regulation reached significance in three podocytes lines from nephrotic syndrome patients (Fig. 7A-B). No effect on SUMO1 or SENP2 modulation was observed using MSC-EVs (not shown). Subsequently, we aimed at confirming the relevance of SUMO1 and/or SENP2 on podocyte function. For this purpose, we performed the permeability assay using CTL-tPOD that had been downregulated for either SUMO1 or SENP2 genes by transfection with siRNA (Fig. 7C). Permeability assay revealed an increased BSA-FITC permeability in CTL-tPOD transfected with SENP2 siRNA either alone or combined with SUMO1 siRNA (Fig. 7D).

## Discussion

The current study provides a novel approach to directly assess the potential of regenerative therapies on patient-derived podocytes with genetic alterations. We demonstrated the efficacy of EVs derived from neonatal renal progenitor cells in improving permeability in podocytes obtained from patients with steroid-resistant nephrotic syndrome. Furthermore, we identified the SUMOylation pathway as a possible common mechanism in all patient-derived cell lines treated with nKPC-EVs.

The availability of human podocytes that accurately replicate human pathologies in culture is currently limited. Recent advancements have allowed the derivation of podocyte-like cells from induced pluripotent stem cells from patients, offering a promising avenue [41]. However, obtaining a purely homogeneous population of podocytes is challenging; typically, only 30–50% of cells in induced pluripotent stem cell cultures exhibit podocyte-like morphology [42]. Another potential source for modeling diseases is the differentiation of urinary renal progenitor cells into podocytes, although this process may involve cell culture and differentiation stimuli [43].

Previous studies have utilized urine as a non-invasive and valuable source to obtain podocytes [11]. In patients with active glomerular diseases, podocytes are shed from the glomerulus in response to local environmental factors. Urine patient-derived podocytes have been demonstrated to be positive for podocyte markers, viable, and capable of growth in culture [11]. Conversely, due to their limited number and limited replication potential in culture, podocytes cannot be obtained from normal subjects’ urine [11]. Of interest, increased podocyturia is observed in the urine of uncomplicated pregnant women [24], making it a valuable source for obtaining healthy control podocytes.

In this context, our in vitro model of glomerular permeability utilizing podocytes directly obtained from patients’ urine holds significant promise for personalized medicine. This study includes podocytes with mutations affecting a slit diaphragm protein (podocin), matrix synthesis components (collagen IV and laminin) and a calcium balance regulator (Phospholipase C epsilon 1). We here showed the feasibility of a patient-specific permeability model that could be used in personalized medicine to evaluate the impact of drugs commonly administered to patients with nephrotic syndrome. Notably, podocytes derived from steroid resistant patients, unresponsive to therapies, also did not respond to the drugs previously reported to modulate permeability [36–38]. An effect on permeability was only observed on the podocyte line from a patient who, despite initially clinically steroid-resistant, exhibited subsequent clinical improvement with minimal proteinuria after 48 months (NS-POD3). In the future, a personalized approach could potentially set the stage for tests capable of predicting patient responses, guiding clinicians in treatment decisions, and avoiding unnecessary immunosuppressive interventions in the management of nephrotic syndrome. It must be however underlined that the short survival of those primary lines only allows a restricted number of tests.

Another potential application of the primary model of the glomerular filtration barrier is the evaluation of innovative biological therapeutics. We here assessed the effect of nKPS-EVs on genetically mutated patient-derived podocytes. Our findings indicate that nKPCs could serve as a valuable source of EVs with regenerative potential. Interestingly, no effect on healthy podocytes was observed, further supporting the importance of disease models to assess therapeutic approaches. Moreover, in consideration to the variability of single line response, the use of different primary cell lines is instrumental to identify a possible nKPC-EV specific mechanism of action.

EVs, in particular those obtained from MSCs, have been previously studied in in vitro and in vivo experimental models of kidney disease [19, 44–48]. Using a millifluidic model of glomerular filtration barrier, we previously demonstrated that MSC-EVs could traverse the basal membrane, reach podocytes and transfer their RNA cargo, resulting in protection against doxorubicin-induced injury [19]. Other studies have underscored the protective roles of MSC-EVs on mouse podocyte lines using a model of diabetic nephropathy [47, 48]. However, the identification of specific mechanisms involved in the effect of EVs on target cells appears quite challenging, in consideration to the multitude of EV cargoes and to the patient-derived cell heterogeneity.

In our approach, we searched for common nKPC-EV-regulated genes at the transcriptomic level. Of interest, SUMO1 and SENP2, its modulator, were identified as the only common genes concordantly regulated in all patient-derived podocyte lines. At variance, no SUMO or SENP2 modulation was detected using MSC-EV treatment. Although we did not identify the factors involved in the specific effect of nKPS-EVs on podocytes due to the complexity of EV cargo, it might be speculated that factors linked to their renal progenitor origin, such as SIX2 expression, could promote SUMO1 and SENP2 expression, known to be relevant in epithelial cell differentiation [22]. This could be supported by data obtained using the EV originating cells, nKSPCs, administered to human kidneys discarded for transplantation. In the study, the *de novo* expression of SIX2 in proximal tubular cells and upregulated regenerative markers, including SOX9, was observed [22].

SUMOylation pathway is considered to be involved in slit diaphragm protein stabilization, thus playing a possible role in the control of glomerular permeability [49]. Further studies will be necessary to better elucidate this point. The activity of SUMO, or SUMOylation, consists in a post-translational modification that alters the function of target proteins and modulates cell processes such as protein stability, localization, and activity [49, 50]. Since SENP2 is a known regulator of SUMO1 [38], it may be indirectly involved in slit diaphragm stabilization as well. This was also confirmed by experiments of SUMO1 and SENP2 downregulation, demonstrating their involvement on podocyte permeability in our in vitro model. Concordantly, Gene Ontology analysis highlighted cell-to-cell adhesion as the primary upregulated biological activity in nKPC-EV treated podocytes. Therefore, the potential effect of EVs derived from kidney progenitors on genetically altered podocytes represents a promising strategy for addressing genetic pathologies currently lacking a therapeutic solution. While this strategy was tested on three podocyte lines in this study, further validation and expansion to a larger patient cohort are warranted.

## Conclusion

In conclusion, we here set up a human in vitro functional model for the analysis of drug response on albumin permeability, thanks to combined effort of clinical, genetic and basic science. Our findings demonstrated the positive impact of nKPC-EVs in improving the function of genetically altered podocytes and identified the effect of EVs on the regulation of SUMOylation, an important pathway for stabilizing podocyte slit diaphragm proteins.

Our data may pave the road for establishing a standard non-invasive in vitro model for the screening of regenerative compounds directly on patient-derived podocytes.

### Electronic supplementary material

Below is the link to the electronic supplementary material.

Supplementary Material 1

Supplementary Material 2

## Data Availability

RNA sequencing data are available in the supplementary data.

## Abbreviations

EVs: Extracellular vesicles
nKPCs: Neonatal kidney progenitor cells
NS-POD: Nephrotic syndrome podocytes
AS-POD: Alport syndrome podocytes
CTL-uPOD: Control urine podocytes
CTL-tPOD: Control tissue podocytes
FCS: Foetal calf serum
BSA: Bovine serum albumin
NTA: Nanoparticle tracking analysis
TEM: Transmission electron microscopy
RT-PCR: Real-time PCR
PE: Phycoerythrin
FITC: Fluorescein isothiocyanate
APC: Allophycocyanin
MPB: MACSPlex buffer
CKD: Chronic kidney disease
SSEA-4: Stage-specific embryonic antigen-4
M-PR: Methylprednisolone
CYCLO: Cyclosporin
TAC: Tacrolimus
RITUX: Rituximab
MSCs: Mesenchymal stromal cells
SUMO1: Small ubiquitin-related modifier 1
SENP2: Sentrin-specific protease 2
SCR: Scramble sequence
LP: Lipofectamine
