# Supplementary material for: Urine-derived podocytes from steroid resistant nephrotic syndrome patients as a model for renal-progenitor derived extracellular vesicles effect and drug screening
Source: J Transl Med. 2024 Aug 14;22:762. doi: 10.1186/s12967-024-05575-z (PMC11323595; doi:10.1186/s12967-024-05575-z)
Supplement: Supplementary file 2 — Supplementary Material 2 [file 12967_2024_5575_MOESM2_ESM.docx]

**Urine-derived podocytes from steroid resistant nephrotic syndrome patients as a model for renal-progenitor derived extracellular vesicles effect and drug screening**

Adele Tanzi^a*^, Lola Buono^a*^, Cristina Grange^b^, Corinne Iampietro^a^, Alessia Brossa^a^, Fanny [Oliveira](https://pubmed.ncbi.nlm.nih.gov/?term=Arcolino+FO&cauthor_id=35913414) Arcolino^c,d^, Maddalena Arigoni^a^, Raffaele Calogero^a^, Laura Perin^e^, Silvia Deaglio^b^, Elena Levtchenko^c,f^, Licia Peruzzi^g^ and Benedetta Bussolati^a^

*^a^Department of Molecular Biotechnology and Health Sciences, University of Turin, Turin, Italy;*

*^b^Department of Medical Sciences, University of Turin, Turin, Italy;*

*^c^Department of Pediatric Nephrology, Emma Children’s Hospital, Amsterdam UMC, The Netherlands;*

*^d^Emma Centrum of Personalized Medicine, Emma Children’s Hospital, Amsterdam UMC, The Netherlands;*

*^e^Department of Urology, Children's Hospital Los Angeles, Los Angeles, California;*

*^f^Department of Development and Regeneration, Cluster Woman and Child, Laboratory of Pediatric Nephrology, KU Leuven, Leuven, Belgium;*

*^g^Pediatric Nephrology, ERKNet Center, Regina Margherita Children’s Hospital, AOU Città della*

*Salute e della Scienza di Torino, Turin, Italy.*

**Supplementary Information**

**Supplementary Figure 1. Podocyte markers expression.**

**
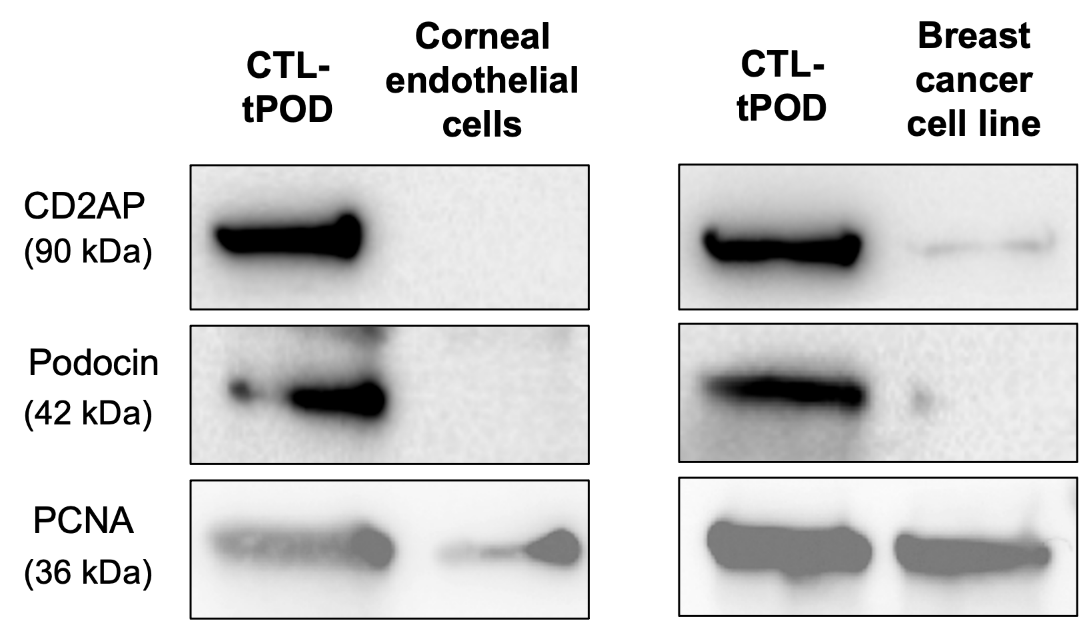
**

***Supplementary Figure 1.*** *Representative images of Western Blot bands of CD2AP and Podocin, two classical podocyte markers. These markers appeared negative in corneal endothelial and breast cancer cells, used as negative controls. PCNA was used as housekeeping.*

**Supplementary Figure 2. RNA-sequencing analysis of urine-derived podocytes.**


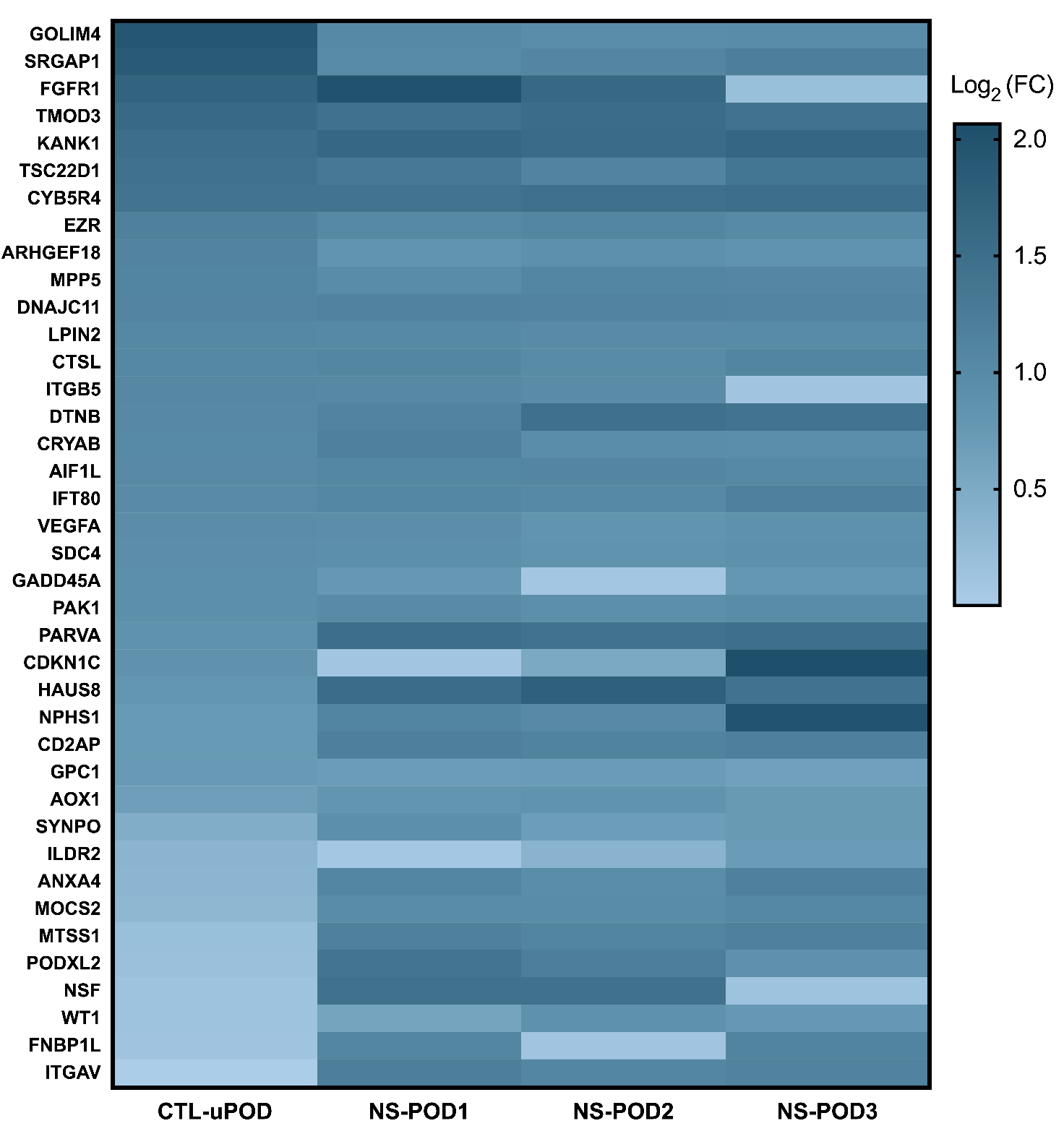


***Supplementary Fig. 2.*** ***RNA-sequencing analysis of urine-derived podocytes.*** *Heatmap of podocyte signatures showing the fold change (FC) of podocyte-specific genes in CTL-uPOD and NS-POD lines with respect to CTL-tPOD.*

**Figure 3. Cell viability after drug treatments.**

**
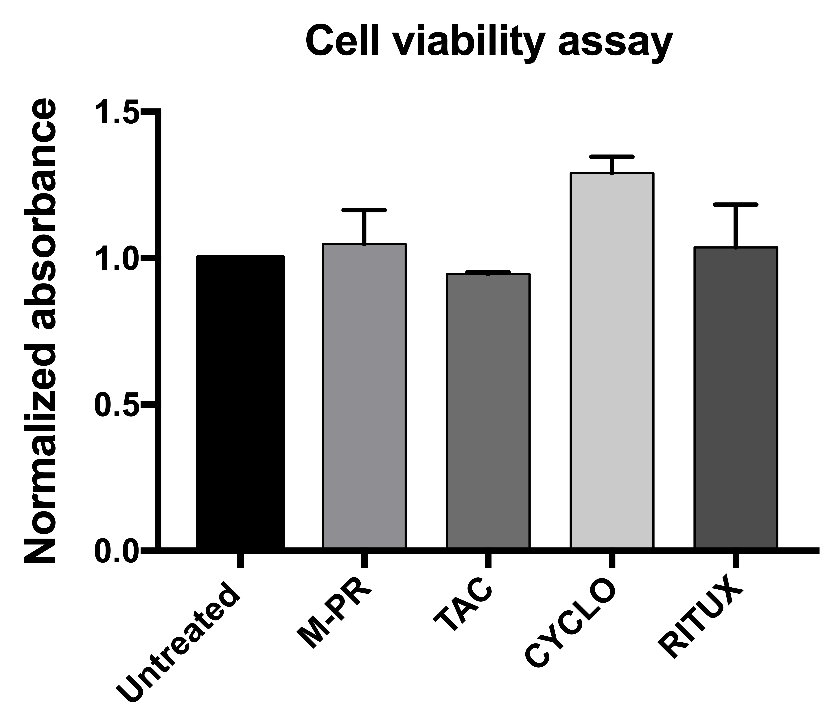
**

**Figure 3. Cell viability after drug treatments.**

*The histogram represents the normalized absorbance after MTT administration of CTL-tPOD treated with different drugs: methylprednisolone (M-PR, 40* μ*g/mL), cyclosporin (CYCLO, 25* μ*g/mL), tacrolimus (TAC 1 ng/mL) and rituximab (RITUX 12* μ*g/mL) for 24 h. Untreated podocytes were used as control, set as 1. Data are expressed as the mean of two experiments ± SD. One-way ANOVA with Dunnett's multiple comparisons test was performed, revealing no significant difference among conditions.*

**Supplementary table**

**Supplementary Table 1.** Primers used for RT-PCR analyses.

|  | Primer forward | Primer reverse |
| --- | --- | --- |
| GAPDH | TGGAAGGACTCATGACCACAGT | CATCACGCCACAGTTTCCC |
| SIX2 | CACAGGTCAGCAACTGGTTCA | GAGCTGCCTAACACCGACTTG |
| FOXD1 | ACCCTGAGCACTGAGATGTC | CCACGTCGATGTCTGTTTCC |
| SUMO1 | GTCAAAGACAGGGTGTTCCAATG | CTTCTTCCTCCATTCCCAGTTCT |
| SENP2 | CATGGCCCACAGGATGAAAT | TGACTTCATCATTGAGCCAGTGA |

**Supplementary Methods**

**Cell viability assay (MTT)**

To analyze cell viability, 7.5 × 10^3^ CTL-tPOD cells/well were seeded in a 96 well plate, left adhere overnight at 33 °C and therefore moved to 37 °C to differentiate. After 10 days at 37 °C, cells were treated for 24 h with different drugs: methylprednisolone (M-PR, 40 μg/mL), cyclosporin (CYCLO, 25 μg/mL), tacrolimus (TAC 1 ng/mL) and rituximab (RITUX 12 μg/mL). Subsequently, 10 μl of MTT (3-(4,5-dimethylthiazol-2-yl)-2,5-diphenyl-2H-tetrazolium bromide) labeling reagent (Merck) were added to each condition and cells were incubated at 37 °C. After 4 h, 10 μl of isopropanol with 0.04 N HCl were added into each well. Within 1 h the 560 nm absorbance of the samples was measured using Promega™ GloMax® Plate Reader (Promega Italia S.r.l).
